# Clinical decision-making style preferences of European psychiatrists: Results from the Ambassadors survey in 38 countries

**DOI:** 10.1192/j.eurpsy.2022.2330

**Published:** 2022-10-21

**Authors:** Martina Rojnic Kuzman, Mike Slade, Bernd Puschner, Elisabetta Scanferla, Zarko Bajic, Philippe Courtet, Jerzy Samochowiec, Celso Arango, Simavi Vahip, Maris Taube, Peter Falkai, Geert Dom, Lubomira Izakova, Bernardo Carpiniello, Marcella Bellani, Andrea Fiorillo, Oleg Skugarevsky, Alma Mihaljevic-Peles, Diogo Telles-Correia, Filipa Novais, Pavel Mohr, Johannes Wancata, Martin Hultén, Eka Chkonia, Judit Balazs, Julian Beezhold, Lars Lien, Goran Mihajlovic, Mirjana Delic, Gabriela Stoppe, Goran Racetovic, Dragan Babic, Ramune Mazaliauskiene, Doina Cozman, Simon Hjerrild, Jana Chihai, William Flannery, Tarja Melartin, Nataliya Maruta, Armen Soghoyan, Philip Gorwood

**Affiliations:** 1 Department of Psychiatry and Psychological Medicine, University Hospital Centre Zagreb and School of Medicine, University of Zagreb, Zagreb, Croatia; 2 Institute of Mental Health, School of Health Sciences, University of Nottingham, Nottingham, United Kingdom; 3 Health and Community Participation Division, Faculty of Nursing and Health Sciences, Nord University, Namsos, Norway; 4 Department of Psychiatry II, Ulm University, Ulm, Germany; 5 ED 450, Université de Paris, Paris, France; 6 GHU Paris Psychiatry and Neuroscience, Clinic of Mental Illnesses & Brain Disorders, 75014, Paris, France; 7 Research Unit “Dr. Mirko Grmek”, Psychiatric Clinic “Sveti Ivan”, Zagreb, Croatia; 8 Department of Emergency Psychiatry and Post Acute Care, Hôpital Lapeyronie, CHU Montpellier, Montpellier, France; 9 Council of National Psychiatric Associations, EPA, Strasbourg, France; 10 Pomeranian Medical University in Szczecin, Szczecin, Poland; 11 Department of Child and Adolescent Psychiatry, Institute of Psychiatry and Mental Health, Hospital General Universitario Gregorio Marañón, School of Medicine, IiSGM, CIBERSAM, Complutense University of Madrid, Madrid, Spain; 12 Affective Disorders Unit, Department of Psychiatry, Ege University Medicine Faculty, Izmir, Turkey; 13 Department of Psychiatry and Narcology, Riga Centre of Psychiatry and Narcology, Riga Stradiņš University, Riga, Latvia; 14 Department of Psychiatry and Psychotherapy, Ludwig-Maximilian-University Munich, Munich, Germany; 15 Collaborative Antwerp Psychiatric Research Institute (CAPRI), University of Antwerp (UAntwerp), Antwerp, Belgium; 16 Department of Psychiatry, Faculty of Medicine, Comenius University in Bratislava, Bratislava, Slovakia; 17 University of Cagliari and Psychiatry Unit, Section of Psychiatry, Department of Medical Sciences and Public Health, University Hospital, Cagliari, Italy; 18 Department of Neurosciences Biomedicine and Movement Sciences, University of Verona, Verona, Italy; 19 EPA Sections, Strasbourg, France; 20 Department of Psychiatry, University of Campania “L. Vanvitelli”, Naples, Italy; 21 Department of Psychiatry and Medical Psychology, Belarusian State Medical University, Minsk, Belarus; 22 Clinica Universitária de Psiquiatria e Psicologia Médica, Faculdade de Medicina, Universidade de Lisboa, Lisbon, Portugal; 23 Clinical Department, National Institute of Mental Health, Klecany, Czechia; 24 Third School of Medicine, Charles University, Prague, Czech Republic; 25 Division of Social Psychiatry, Medical University of Vienna, Vienna, Austria; 26 Psychiatric Neuromodulation Unit (PNU), Department of Clinical Sciences, Lund, Faculty of Medicine, Lund University, Lund, Sweden; 27 Department of Psychiatry, Tbilisi State Medical University, Tbilisi, Georgia; 28 Department of Developmental and Clinical Child Psychology, Institute of Psychology, Eötvös Loránd University, Budapest, Hungary; 29 Department of Psychology, Oslo New University College, Oslo, Norway; 30 Norwich Medical School, University of East Anglia, Norwich, United Kingdom; 31 Norwegian National Advisory Unit on Concurrent Substance Abuse and Mental Health Disorders, Innlandet Hospital Trust, Hamar, Norway; 32 Clinic for Psychiatry, University of Kragujevac, Kragujevac, Serbia; 33 Center for Treatment of Drug Addiction, University Psychiatric Clinic, Ljubljana, Slovenia; 34 MentAge Basel and Medical Faculty, University of Basel, Basel, Switzerland; 35 Community Mental Health Center, Health Center Prijedor, Prijedor, Bosnia and Herzegovina; 36 Department of Psychiatry, University Hospital Mostar, Mostar, Bosnia and Herzegovina; 37 School of Medicine, University of Mostar, Mostar, Bosnia and Herzegovina; 38 Psychiatric Clinic, Lithuanian Health Sciences University Kaunas Hospital, Lithuanian Health Sciences University, Kaunas, Lithuania; 39 Iuliu Hatieganu University of Medicine and Pharmacy, Cluj-Napoca, Romania; 40 Psychosis Research Unit, Aarhus University Hospital, Aarhus, Denmark; 41 Department of Mental Health, Medical Psychology and Psychotherapy, Medical Psychology State Medical and Pharmaceutical University “Nicolae Testemitanu” from Republic of Moldova, Kishinev, Moldova; 42 Department of Adult Psychiatry, Mater Misericordiae University Hospital, Dublin, Ireland; 43 Department of Psychiatry, Helsinki University Central Hospital, Helsinki, Finland; 44 State Institution “Institute of Neurology, Psychiatry and Narcology of the NAMS of Ukraine”, Kharkiv, Ukraine; 45 Psychosocial Recovery Center, Yerevan State Medical University after Mkhitar Heraci, Yerevan, Armenia

**Keywords:** Clinical decision-making, Europe, mental health, professional-patient relations, psychiatry, shared decision-making

## Abstract

**Background:**

While shared clinical decision-making (SDM) is the preferred approach to decision-making in mental health care, its implementation in everyday clinical practice is still insufficient. The European Psychiatric Association undertook a study aiming to gather data on the clinical decision-making style preferences of psychiatrists working in Europe.

**Methods:**

We conducted a cross-sectional online survey involving a sample of 751 psychiatrists and psychiatry specialist trainees from 38 European countries in 2021, using the Clinical Decision-Making Style – Staff questionnaire and a set of questions regarding clinicians’ expertise, training, and practice.

**Results:**

SDM was the preferred decision-making style across all European regions ([central and eastern Europe, CEE], northern and western Europe [NWE], and southern Europe [SE]), with an average of 73% of clinical decisions being rated as SDM. However, we found significant differences in non-SDM decision-making styles: participants working in NWE countries more often prefer shared and active decision-making styles rather than passive styles when compared to other European regions, especially to the CEE. Additionally, psychiatry specialist trainees (compared to psychiatrists), those working mainly with outpatients (compared to those working mainly with inpatients) and those working in community mental health services/public services (compared to mixed and private settings) have a significantly lower preference for passive decision-making style.

**Conclusions:**

The preferences for SDM styles among European psychiatrists are generally similar. However, the identified differences in the preferences for non-SDM styles across the regions call for more dialogue and educational efforts to harmonize practice across Europe.

## Introduction

Decision-making includes context (information and preferences), the actual process of decision-making and its evaluation, and the outcome [1–3]. Three types of decision-making have been proposed to characterize the degree of patient involvement: passive or paternalistic (decision is made by staff and patient consents), shared (information is shared and decision jointly made), and active (staff informs and patient decides) [4, 5]. Although the concept of shared clinical decision-making (SDM) has evolved over time [6], we support the definition of SDM as “a process in which clinicians and patients work together to select tests, treatments, management or support packages, based on clinical evidence and the patient’s informed preferences; it involves the provision of evidence-based information about options, outcomes and uncertainties, together with decision support counselling and a system for recording and implementing patients’ informed preferences” [7].

Because of its collaborative aspect, SDM is becoming the preferred approach in clinical practice including diagnosis, treatment, and evaluation, and is strongly advocated for by service users, service providers, and policymakers [8, 9]. It has become an integral part of value‐based health care, which is a health care delivery model organized around patients’ needs and outcomes while optimizing resource utilization [10–12].

However, while widely recommended [9] by patient organizations, healthcare professionals, policy makers, and also endorsed by the general public, SDM is still unevenly applied by medical professionals [13, 14] and there is a lack of proper adoption strategies [15]. In the field of mental health, the implementation of SDM remains limited, although the components of SDM have been elucidated, and decision-support tools are available [9].

Identified challenges to a systematic implementation of SDM include the lack of required training [9], non-adoption of Patient Reported Experience Measures (PREMs) and Patient Reported Outcome Measures (PROMs) in clinical practice [8], workplace-related challenges/specificities (e.g., working at large hospitals and public settings, working with in-patients, having limited time for clinical visits) [16], the predominant method of delivered treatment (e.g., biological versus psychotherapy) and possibly economic aspects (e.g., the cost of visits and portion of reimbursement for patients) [11]. However, as a European-wide association, we were interested in analyze whether there are regional differences in the SDM practices between mental health care professionals working in Europe. We assumed that different socio-cultural contexts of the European regions affected the general concepts of mental health and the organization of mental health care services and shaped the preferences for clinical decision-making styles of mental health care professionals. Thus, the European Psychiatric Association (EPA) undertook this study with the primary aim to identify the preferences for clinical decision-making styles of psychiatrists working in different European regions (Central and Eastern, Northern, Southern, and Western Europe). We additionally analyzed the preferences for passive versus other styles in our sample, as the passive style is associated with the lowest level of patient involvement in the decision-making process.

## Methods

### Study design, settings, and participants

The EPA approached psychiatrists working in Europe, who were associated with the EPA community, including individual members of the EPA and its Member Associations, and attendees of the last 10 congresses of the EPA. In 2020, they were offered the opportunity to become “EPA Ambassadors” and to participate in EPA surveys. We initially sent an invitation email to previous EPA congress participants, comprising around 5,000 individuals. Subsequently, the Council of National Psychiatric Associations, the Board, and the EPA Sections were asked to distribute the invitations among their members. Responses were collected from April to December 2021, using an online questionnaire. The study was open to all mental health professionals (psychiatrists, psychiatry specialist trainees, psychologists, social workers, and nurses) working in Europe. The authors assert that all procedures contributing to this work comply with the ethical standards of the relevant national and institutional committees on human experimentation and with the Helsinki Declaration of 1975, as revised in 2008 and 2013 [17]. The study was approved by the Ethical Committee of the Zagreb University Hospital Centre, No. 02/013-JG.

### Materials/data sources

Decision-making was evaluated using the standardized questionnaire Clinical Decision-Making Style – Staff (CDMS-S) [18–20] which measures “Participation in Decision-Making” via two subscales (Sections A and B) with all items rated on five-point Likert scales. Section A comprises six items (rated from “strongly disagree” to “strongly agree”) to indicate general preferences for decision-making in routine mental health services. Section B comprises nine items (rated from “service user” to “me”) to indicate specific preferences for decision-making in relation to three clinical vignettes (dealing with work, medication side effects, and medication in general). Items 1, 2, 3, and 5 are reversed. The Participation in Decision-Making sub-scale is the prorated mean of all items in Sections A and B and can be calculated when at least 12 of the 15 items have been rated. It ranges from 0 to 4, with a higher score indicating a higher preference by the clinician for active service-user participation in decision making.

Additional questions included data on socio-demographics (age, sex, and city size) and clinicians’ type of expertise, training and practice (time since they qualified as consultant psychiatrists, subspecialty, working position, type of practice, clinical setting, and duration of visits/appointments), and place of work (specifically, the use of SDM and previous use of PREMs and PROMs).

### Variables and outcomes

The primary outcome was the total score on the CDMS-S questionnaire. Possible moderators, whose effects we controlled with multivariable analyses, were sex, age, city size, profession, practice, treating mainly in-or outpatients, subspecialty, providing psychotherapy, time since they qualified as consultant psychiatrists, cost of visit, and average frequency of clinical appointments with each patient. The secondary outcomes were a prevalence of preference for a passive decision-making style, defined as CDMS-S score < 1.5, promotion of shared decision-making style in the department/institution, and usage of PROMs/PREMs. The cut-off value for the active CDMS was 2.5.

### Statistical analysis

To correct imbalances in the regional distribution of the sample compared to the regional distribution of the total European psychiatrists’ population, that is, to make the sample of psychiatrists and psychiatry trainees’ statistics more representative for the target psychiatrists’ population parameters, we used a poststratification of non-response weights on the country level. We calculated these by dividing the proportion of each country’s population in the total European population by the proportion of each country sample in the total sample (Supplementary Table 1). We did a multiple imputation of missing data and presented the details in the Supplementary Material. We categorized countries into three regions according to EuroVoc (Supplementary Material): central and eastern Europe (CEE), northern and western Europe (NWE), and southern Europe (SE). We grouped NWE into a single group, because the sample size from Northern Europe was only 46. We assessed the reliability and unidimensionality of CDMS-S, and have explained these procedures in the Supplementary Material. As the introductory analysis of our primary outcome, the total CDMS-S score, we conducted a series of bivariable linear regressions and presented unstandardized regression coefficients with 95% confidence interval (CI). As the main analysis, we used a single multivariable linear regression model where we included all variables simultaneously. We analyzed the secondary outcome, prevalence of preferred clinical decision-making style, using multinomial regression analysis where we presented relative risk ratios (RRRs) as the standardized effect size measures. We corrected statistical significances for multiple testing using the Benjamini–Hochberg procedure with the false discovery rate (FDR) set at <5%. We conducted analyses using StataCorp (StataCorp LLC, College Station, TX) [21].

The manuscript is written in accordance with the STROBE guidelines (Supplementary Material) for reporting cross-sectional studies [22].

## Results

### Participant characteristics

The online survey was answered by 919 participants from 38 European countries. We excluded 27 (3.0%) psychologists, 14 (1.5%) other mental health professionals, and 10 (1.1%) participants with unknown profession, leaving 738 (81.2%) psychiatrists and 130 (14.3%) psychiatry trainees, a total of 868 participants in the sample from the target population. Complete data on all 15 CDMS-S items were missing from 112/868 (12.9%) responses, and they were excluded from the sample because no reliable imputation was possible given the moderate associations of other variables with the 15 items of our primary outcome. We further excluded five participants with incomplete data on 14 CDMS-S items (*n* = 2), 13 items (*n* = 1), and 10 items (*n* = 2). Thus, the number of participants included in the final analysis was 751/868 (86.5%).

In the final sample of 751 participants, 322 (42.9%) were from CEE, 273 (36.4%) were from NWE, and 156 (20.8%) were from SE (Table 1). The regional structure of the final sample was markedly different from the regional structure of the total target population of European psychiatrists (Supplementary Figure 1). CEE subpopulation was overrepresented, while SE subpopulations were underrepresented. We corrected these imbalances using poststratification weighting (Supplementary Material). Samples from the three European regions were relatively comparable for sex, with 357 (47.8%) women. Most participants were psychiatrists, and 108 (14.4%) were psychiatry specialist trainees. Participants from NWE were more often from larger cities. Samples from the three European regions were well balanced in terms of age and subspecialty.

**Table 1.** Participant characteristics by region (unweighted data).

	Whole sample (*n* = 751)	European regions		
		Central and eastern (*n* = 322)	Northern and western (*n* = 273)	Southern (*n* = 156)
Sex, *n* (%)				
Women	357 (47.8)	162 (50.5)	118 (43.5)	77 (49.7)
Men	390 (52.2)	159 (49.5)	153 (56.5)	78 (50.3)
Age, *n* (%)				
18–29	80 (10.7)	38 (11.8)	18 (6.6)	24 (15.4)
30–39	222 (29.6)	96 (29.8)	74 (27.2)	52 (33.3)
40–49	181 (24.1)	79 (24.5)	69 (25.4)	33 (21.2)
50–59	167 (22.3)	73 (22.7)	68 (25.0)	26 (16.7)
60+	100 (13.3)	36 (11.2)	43 (15.8)	21 (13.5)
City size, *n* (%)				
≤100,000	164 (21.9)	65 (20.2)	85 (31.3)	14 (9.0)
>100,000	584 (78.1)	256 (79.8)	187 (68.8)	141 (91.0)
Profession, *n* (%)				
Psychiatrist	643 (85.6)	275 (85.4)	243 (89.0)	125 (80.1)
Psychiatry specialist trainee	108 (14.4)	47 (14.6)	30 (11.0)	31 (19.9)
Practice, *n* (%)				
Public	466 (62.3)	177 (55.1)	190 (69.9)	99 (63.9)
Mixed	146 (19.5)	77 (24.0)	31 (11.4)	38 (24.5)
Private	99 (13.2)	54 (16.8)	29 (10.7)	16 (10.3)
Community setting	37 (4.9)	13 (4.0)	22 (8.1)	2 (1.3)
Patients, *n* (%)				
Mainly outpatients	469 (62.8)	189 (58.9)	163 (60.1)	117 (75.5)
Mainly inpatients	278 (37.2)	132 (41.1)	108 (39.9)	38 (24.5)
Subspecialty, *n* (%)				
Specialized	485 (64.7)	199 (61.8)	188 (68.9)	98 (63.2)
Unspecialized	265 (35.3)	123 (38.2)	85 (31.1)	57 (36.8)
Subspecialty, *n* (%)				
Mood and anxiety disorders	152 (20.3)	74 (23.0)	56 (20.5)	22 (14.2)
Psychosis	126 (16.8)	57 (17.7)	46 (16.8)	23 (14.8)
Child psychiatry	68 (9.1)	33 (10.2)	15 (5.5)	20 (12.9)
Addiction	47 (6.3)	18 (5.6)	20 (7.3)	9 (5.8)
Other	92 (12.3)	17 (5.3)	51 (18.7)	24 (15.5)
Unspecialized	265 (35.3)	123 (38.2)	85 (31.1)	57 (36.8)
Providing psychotherapy	315 (42.0)	124 (38.5)	120 (44.0)	71 (45.8)
Time since specialist psychiatric qualification (years), median (IQR)	13 (5; 25)	15 (5; 25)	14 (5; 26)	10 (4; 24)
Average frequency of clinical appointments with each patient, *n* (%)				
Several times a week	113 (15.3)	66 (20.8)	28 (10.5)	19 (12.3)
Several times a month	195 (26.4)	84 (26.5)	85 (31.8)	26 (16.8)
Once a month	309 (41.8)	144 (45.4)	98 (36.7)	67 (43.2)
Less frequent	122 (16.5)	23 (7.3)	56 (21.0)	43 (27.7)
Duration of visit (min), median (IQR)	35 (30–45)	31 (30–45)	40 (30–50)	30 (20–45)

*Note*: Data were presented as the number (percentage) of participants unless stated otherwise.

*Note*: Data were missing for sex in 4 (0.5%), age in 1 (0.1%), city size in 3 (0.4%), practice in 3 (0.4%), patients in 4 (0.5%), subspecialty in 1 (0.1%), time since specialist psychiatry qualification in 10 (1.3%), cost of visit in 271 (361%), and average frequency of clinical appointments with each patient in 12 (1.6%) participants.

*Abbreviation*: IQR, interquartile range.

### Primary outcome data

**Figure 1.**
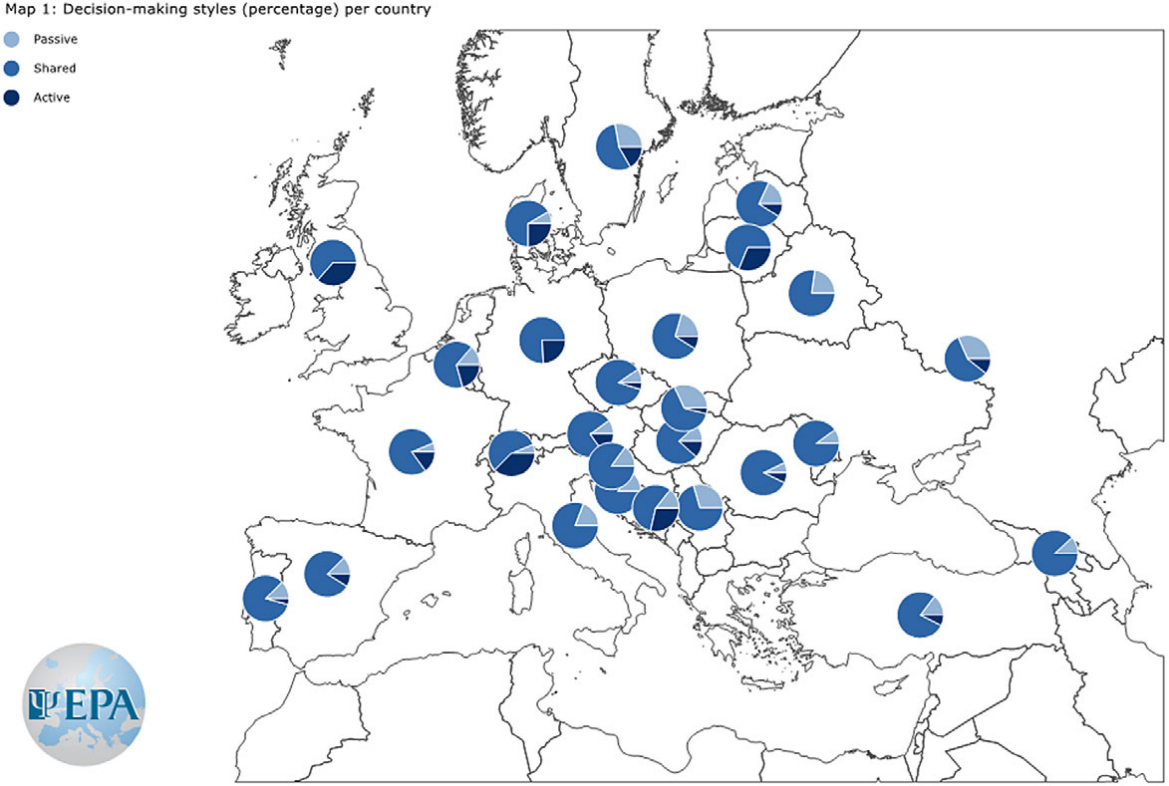
Shared clinical decision-making (SDM) style preferences in European countries with at least 10 study participants.

**Figure 2.**
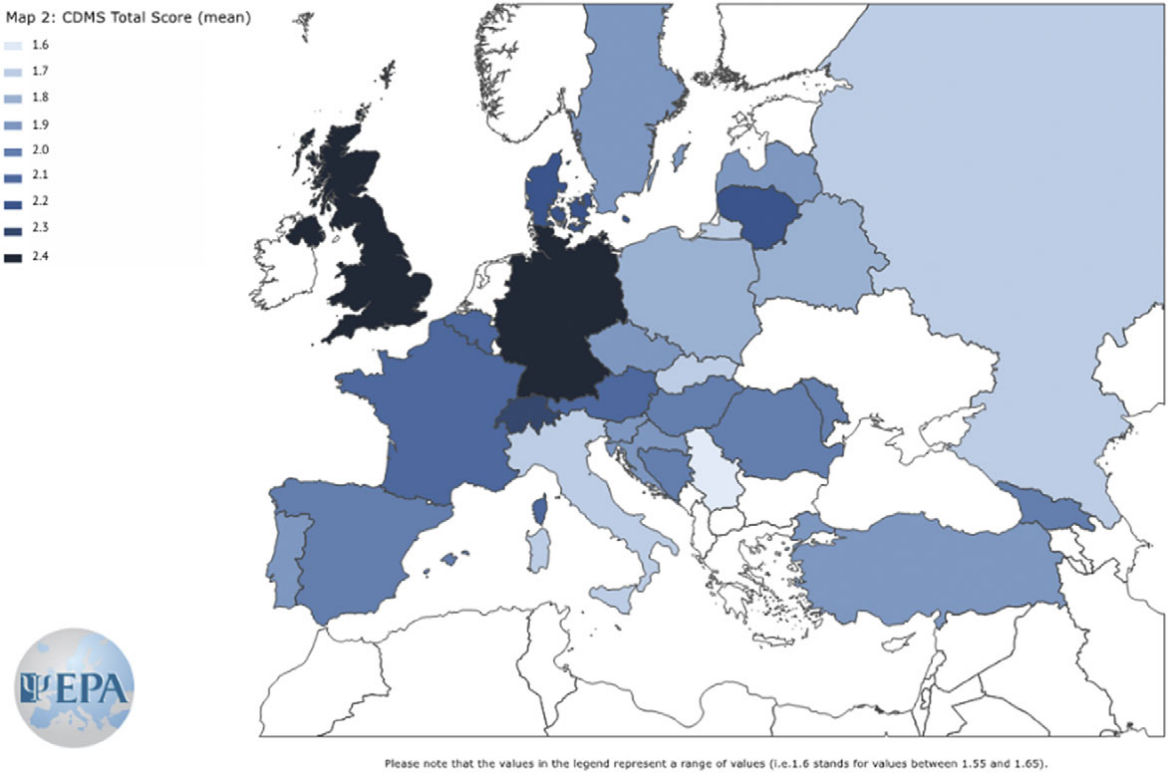
Clinical Decision-Making Style – Staff (CDMS-S) total scores in countries with at least 10 study participants.

Reliability of CDMS-S was acceptable, McDonald’s *ω* = 0.77 (95% CI 0.75; 0.80), but CDMS in this usage was not unidimensional (Supplementary Material). Overall, 46/751 (6.1%) of participants had some missing data on items of the CDMS Section A but no missing data on Section B, and 35/751 (4.7%) participants had missing data on some items of the CDMS Section B but complete data on Section A. These data were multiply imputed as described in the Supplementary Material. With poststratification weighting for particular countries’ sample sizes and multiple imputation of missing data, the mean CDMS-S score was 2.07 (95% CI 2.02; 2.13) (Table 2). We observed the lowest (most passive) CDMS-S score in CEE. The mean score in SE was non-significantly higher (OLS regressions: ∆ = 0.04; 95% CI −0.11; 0.18; *p* = 0.612; FDR > 5%). We observed the highest total CDMS-S score (preference for a more active style) in NWE. Both the CEE and SE CDMS-S total scores were significantly lower than in NWE (OLS regressions: ∆ = −0.44; 95% CI −0.57; −0.31; *p* < 0.001; FDR > 5%; ∆ = −0.40; 95% CI −0.51; −0.30; *p* < 0.001; FDR > 5%, respectively). The total CDMS-S score was 24.3% lower in CEE, and 21.6% lower in SE than in NWE. Total CDM scores and the preferred SDM styles in European countries are shown in Figures 1 and 2.

**Table 2.** Clinical decision-making style preference and its promotion in departments/institutions; imputed missing data and poststratification weights.

	Whole sample (*n* = 751)	European regions		
		Central and eastern (*n* = 322)	Northern and western (*n* = 273)	Southern (*n* = 156)
CDMS-S total score	2.07 (2.02; 2.13)	1.81 (1.69; 1.93)	2.25 (2.19; 2.32)	1.85 (1.76; 1.94)
Section A^a^	2.27 (2.20; 2.34)	1.88 (1.75; 2.00)	2.51 (2.42; 2.61)	2.03 (1.90; 2.16)
Section B^b^	1.95 (1.89; 2.00)	1.77 (1.64; 1.91)	2.09 (2.02; 2.15)	1.74 (1.65; 1.82)
Clinical decision-making style preference, % (95% CI)				
Passive	10 (8; 13)	22 (13; 32)	3 (2; 5)	17 (9; 25)
Shared	73 (68; 79)	69 (59; 80)	72 (65; 80)	80 (71; 88)
Active	16 (11; 21)	8 (2; 15)	24 (17; 31)	3 (0; 7)
Promotion of shared CDMS in department/institution, % (95% CI)				
Officially emphasized	39 (33; 45)	39 (27; 50)	43 (34; 51)	29 (19; 39)
Promoted with incentives	5 (2; 8)	8 (1; 15)	4 (0; 8)	3 (0; 7)
Promoted with training	35 (29; 41)	27 (17; 38)	38 (30; 46)	36 (25; 46)
Not presented	21 (17; 25)	26 (16; 37)	15 (11; 20)	33 (23; 42)
Usage of patient reported experience/outcome measures, % (95% CI)				
Frequently	28 (23; 33)	18 (10; 27)	36 (28; 44)	18 (10; 26)
Sometime	24 (19; 29)	24 (16; 33)	24 (17; 31)	25 (16; 35)
Rarely	21 (16; 25)	18 (11; 25)	20 (13; 26)	27 (18; 35)
Never	27 (22; 31)	39 (29; 50)	21 (16; 26)	30 (21; 39)

*Note*: Data were presented as mean (95% confidence interval) unless stated otherwise.

*Abbreviations*: CDMS, clinical decision-making style; CI, confidence interval.

a Section A (six items): General preferences for decision-making in routine mental health services.

b Section B (nine items): Specific preferences for decision-making in relation to three clinical vignettes (work, medication side effects, and medication in general).

### Main results

The total CDMS-S score in NWE was statistically significantly higher (more active) than in CEE even after adjustment for sex, age, city size, profession, practice, mainly in- or outpatients, subspecialty, providing psychotherapy, time since qualification as a consultant psychiatrist, cost of visit, and average frequency of clinical appointments with each patient (Table 3).

**Table 3.** Mean CDMS-S score by participant characteristics; imputed missing and weighted data.

	Mean (95% CI)	Multivariable analysis	
		*b* (95% CI)	*p*
Region			
Central and Eastern	1.81 (1.69; 1.93)	1	
Northern and Western	2.25 (2.19; 2.32)	0.32 (0.21; 0.43)	<0.001^a^
Southern	1.85 (1.76; 1.94)	−0.02 (−0.15; 0.11)	0.751
Sex			
Women	2.05 (1.96; 2.14)	1	
Men	2.09 (2.02; 2.17)	0.02 (−0.08; 0.12)	0.717
Age			
18–29	1.78 (1.61; 1.96)	1	
30–39	2.01 (1.90; 2.11)	0.15 (−0.08; 0.38)	0.190
40–49	2.04 (1.93; 2.15)	0.13 (−0.12; 0.38)	0.301
50–59	2.26 (2.17; 2.35)	0.26 (−0.00; 0.53)	0.053
60+	2.10 (1.94; 2.26)	0.08 (−0.25; 0.41)	0.648
City size			
≤100,000	2.17 (2.05; 229)	1	
>100,000	2.05 (1.99: 2.11)	−0.05 (−0.17; 0.08)	0.453
Profession			
Psychiatrist	2.08 (2.02; 2.14)	1	
Psychiatry specialist trainee	2.03 (1.92; 2.15)	0.09 (−0.06; 0.24)	0.245
Practice			
Public	2.15 (2.08; 2.22)	1	
Mixed	1.88 (1.77; 1.99)	−0.22 (−0.35; −0.10)	<0.001^a^
Private	1.89 (1.72; 2.07)	−0.21 (−0.41; −0.02)	0.034^a^
Community setting	2.23 (2.10; 2.35)	0.07 (−0.25; 0.11)	0.435
Patients			
Mainly outpatients	2.08 (2.01; 2.15)	1	
Mainly inpatients	2.07 (1.98; 2.16)	−0.12 (−0.24; 0.01)	0.071
Subspecialty			
Unspecialized	2.09 (2.00; 2.18)	1	
Specialized	2.07 (2.00; 2.13)	−0.02 (−0.13; 0.08)	0.679
Subspecialty			
Mood and anxiety disorders	2.01 (1.89; 2.14)	n.u.	
Psychosis	1.99 (1.84; 2.14)		
Child psychiatry	2.00 (1.81; 2.19)		
Addiction	2.04 (1.85; 2.22)		
Other	2.24 (2.10; 2.38)		
Unspecialized	2.09 (1.98; 219)		
Providing psychotherapy			
No	2.09 (2.02; 2.17)	1	
Yes	2.05 (1.95; 2.14)	0.01 (−0.08; 0.11)	0.794
Time since specialist psychiatric qualification (years)	–	0.00 (−0.00; 0.01)	0.217
Average frequency of clinical appointments with each patient			
Several times a week	2.05 (1.88; 2.22)	1	
Several times a month	2.12 (2.02; 2.23)	0.00 (−0.17; 0.17)	0.984
Once a month	2.01 (1.93; 2.08)	−0.09 (−0.25; 0.08)	0.293
Less frequent	2.15 (1.99; 2.31)	0.03 (−0.22; 0.17)	0.811
Duration of visits (min)	–	n.u.	

*Abbreviations*: *b*, unstandardized ordinary least square regression coefficient; CDMS-S, Clinical Decision-Making Style – Staff questionnaire; CI, confidence interval; n.u., not used in the multivariable analysis; *p*, statistical significance of the regression coefficients; mean could not be calculated because the variable is numeric.

a FDR < 5%.

Participants from NWE were significantly more likely to prefer a shared decision-making style, compared to participants from CEE (multinomial logistic regression: RRR = 6.76; 95% CI 3.17; 14.40; *p* < 0.001; FDR < 5%), or to prefer an active decision-making style, rather than passive (multinomial logistic regression: RRR = 18.64; 95% CI 6.60; 52.69; *p* < 0.001; FDR < 5%) (Table 3). Participants from NWE were more likely to prefer a shared decision-making style instead of a passive one, compared to participants from SE (multinomial logistic regression: RRR = 4.42; 95% CI 2.09; 9.35; *p* < 0.001; FDR < 5%), and to prefer an active decision-making style instead of a passive one (multinomial logistic regression: RRR = 36.29; 95% CI 10.72; 122.85; *p* < 0.001; FDR < 5%). Participants working in mixed (RRR = 1.88; 95% CI 1.77; 1.99, *p* < 0.001) and private settings (RRR = 1.89; 95% CI 1.72; 2.07, *p* = 0.034) had significantly higher preference for passive clinical decision making (CDM) versus shared decision-making compared to those in public settings, or those working in community mental health services, where the highest DMS scores were observed (Table 3). In the multivariable, adjusted analysis of passive versus other decision-making style, participants from NWE compared to participants from CEE, and psychiatric trainees compared to psychiatrists, had significantly lower odds for preferring a passive style, while work setting, namely working mainly with inpatients compared to working with outpatients, significantly increased the odds for preferring a passive style (Table 4).

**Table 4.** Binary logistic regression of preferred passive Clinical Decision Making Style – Staff questionnaire (CDMS-S < 1.5); imputed missing and weighted data (*n* = 751).

	Percent (95% CI) with preferred passive style	Multivariable analysis	
		OR (95% CI)	*p*
Region			
Central and Eastern	22 (13; 32)	1	
Northern and Western	3 (2; 5)	0.14 (0.06; 0.32)	< 0.001^a^
Southern	17 (9; 25)	1.01 (0.46; 2.24)	0.975
Sex			
Women	10 (6; 15)	1	
Men	11 (7; 15)	1.23 (0.59; 2.54)	0.583
Age			
18–29	22 (6; 37)	1	
30–39	12 (5; 18)	0.40 (0.10; 1.64)	0.205
40–49	10 (4; 17)	0.46 (0.09; 2.39)	0.355
50–59	6 (3; 9)	0.33 (0.05; 2.16)	0.245
60+	10 (3; 17)	0.67 (0.08; 5.60)	0.713
City size			
≤100,000	9 (4; 13)	1	
>100,000	11 (7; 14)	0.90 (0.38; 2.10)	0.804
Profession			
Psychiatrist	11 (8; 14)	1	
Psychiatry specialist trainee	7 (3; 11)	0.26 (0.08; 0.89)	0.031^a^
Practice			
Public	15 (9; 21)	1	
Other	8 (5; 11)	0.55 (0.26; 1.17)	0.123
Patients			
Mainly outpatients	9 (6; 13)	1	
Mainly inpatients	12 (7; 18)	2.50 (1.15; 5.43)	0.021^a^
Subspecialty			
Unspecialized	9 (6; 13)	1	
Specialized	11 (7; 15)	1.12 (0.60; 2.10)	0.715
Subspecialty			
Mood and anxiety disorders	12 (5; 20)	n.u.	
Psychosis	13 (3; 22)		
Child psychiatry	16 (1; 31)		
Addiction	11 (0; 22)		
Other	6 (0; 12)		
Unspecialized	9 (6; 13)		
Time since specialist psychiatric qualification (years)	–	0.98 (0.93; 1.05)	0.610
Average frequency of clinical appointments with each patient			
Several times a week	15 (4; 25)	1	
Several times a month	9 (4; 14)	0.71 (0.27; 1.85)	0.481
Once a month	10 (5; 15)	0.82 (0.33; 2.04)	0.668
Less frequent	11 (4; 18)	1.24 (0.40; 3.79)	0.709
Duration of visit (min)	–		

*Abbreviations*: CI, confidence interval; OR, odds ratio for the passive clinical decision-making style; n.u., not used in the multivariable analysis; *p*, statistical significance of the difference of OR from 1.00; percentage could not be calculated because the variable is numeric.

a FDR < 5%.

### Other analyses: promotion of SDM and the use of PREMs and PROMs in departments/institutions across Europe

The only statistically significant regional difference in the promotion of SDM style was a higher rate of support for SDM, including training in NWE countries, than in CEE ones (multinomial logistic regression: RRR to not be presented = 2.34; 95% CI 1.10; 5.01; *p* = 0.028; FDR < 5%) (Table 2). PREMs/PROMs are frequently used by 28% (95% CI 23; 33) of participants, and never used in 27% (95% CI 22; 31) (Table 2). Statistically significant regional differences in PREMs/PROMs usage were observed between NWE and both, CEE and SE. In NWE, a significantly higher proportion of participants claimed they use PREMs/PROMs frequently (36% [95% CI 28%; 44%]) than in CEE (18% [95% CI 10%; 27%]) or CE (18% [95% CI 10%; 26%]). The RRRs for using PREMs/PROMs frequently as compared to never using them were RRR = 3.77 (95% CI 3.70; 95% CI 1.74; 7.84; *p* = 0.001; FDR < 5%) between NWE and CEE, and RRR = 2.87 (95% CI 1.38; 5.96; *p* = 0.005; FDR < 5%) between NWE and SE. The difference in usage between CEE and SE was not significant (*p* = 0.575; FDR > 5%).

## Discussion

### Preferences for SDM across Europe

In this study, we investigated the preferred decision-making styles of clinicians in mental health services across the regions of Europe. Overall, we found that SDM was the preferred decision-making style across all three European regions, with approximately 73% of decisions being rated as SDM on average. Although “preferences” may indicate a higher percentage of SDM in comparison with its actual adoption in clinical practice [23], these results are in line with a recent study from Europe where clinicians predominantly used SDM in clinical encounters (shared = 78% versus not shared = 22%) [24]. Of note, that study was done in six European countries, with most participants (88%) coming from only four countries (that according to our study categorization would fall within the NWE group). Optimistically, these data also suggest an increasing preference for SDM compared to earlier studies [24, 25], although their sample comprised data on the use of SDM among patients with severe mental illness only.

However, we found significant differences in the preferred decision-making styles, when comparing the non-SDM style preferences between the three European regions. The differences were driven by the preferences for active and passive decision-making styles. Overall, psychiatrists working in NWE countries more often preferred shared and active decision-making styles rather than passive styles when compared to other European regions, especially to the CEE. In line with this finding, working in NWE countries decreased the preferences for passive style (versus others) compared to CEE countries. As SDM is the preferred decision-making style, both passive and active may be regarded as (not recommendable) extremes in most clinical situations. However, it should be noted that the passive style is associated with the lowest degree of patient involvement in the decision-making process, which probably makes it the least preferable. When users of mental health services and their families are asked about this, they stress how important it is for them to have the liberty to make decisions [26].

These results appear to be related to a complex interplay of historical, cultural, and socioeconomic factors shaping general mental health care in the three European regions. These factors certainly include the role of psychiatrists regarding the human rights of persons with mental illness (e.g., the oppression of the human rights of psychiatric service-users imposed by the authorities which were reported in the countries of the CEE before 1990) [27], the status of psychiatry/mental illness within the community (e.g., high levels of stigma in the CEE countries) [28], as well as government policies and financial support for the development of mental health services within countries. For example, over the last few decades Northern and Western high-income countries introduced a large array of multidisciplinary community-based services for people with mental health problems transforming mental health care services from predominantly hospital-based into recovery-oriented community-based care models, promoting social inclusion and empowerment [29]. Recovery-oriented care placed the functional recovery of persons, instead of symptom reduction, as a goal of treatment and demanded services that supported these human rights (including supported employment and housing) [30]. This process is only now beginning in the majority of the Commonwealth of Independent States (which incorporates most of the former Soviet Union’s member states), eight countries from south-eastern Europe, and seven non-EU high-income countries [28, 31].

### The promotion and use of SDM, PREMs, and PROMS across Europe

When it comes to the educational differences between the three European regions, we found that official promotion of SDM with staff training, as well as the use of PREMs and PROMs in evaluation of outcomes was higher in NWE compared to the CEE, possibly suggesting that official endorsement and education of staff in SDM and the relevance of using PROMs and PREMs and the value‐based health care approach may affect their clinical attitude towards patients. However, it might be that lower PROMs/PREMs usage and the lower preference of SDM are both underpinned by the same moderator of cultural aspects.

### Factors associated with preference for DMS across Europe

Two other factors increased the likelihood of preferring a passive decision-making style, namely being a psychiatrist and working mainly with inpatients. First, being a psychiatrist compared to being a trainee increased the likelihood of passive SDM. Interestingly, this is discordant with the results from a European multicentric study, where it was found that trainee status in psychiatry reduced the likelihood of adopting SDM in contrast to being a psychiatrist or other professional [24].

The difference in our findings from the study by Luciano et al. [24] may possibly be explained by sample differences. As mentioned earlier, the study by Luciano et al. [24] included a sample of predominantly mental health professionals from the NWE group of countries only, while our sample included distinct European regions. Countries from the CEE regions underwent dramatic societal changes since 1990, which may have also created a greater sociocultural gap between generations compared to the rest of Europe. For example, younger generations of professionals in CEE countries may be more likely to prefer a more active decision-making approach, as compared to the older generation of psychiatrists who may be more prone to the traditional paternalistic approach. This may not apply to the NWE countries, where the 1990s changes were not associated with system changes and the SDM principles were adopted much earlier in recovery-oriented psychiatric services, and thus the differences between the older versus younger generations in this instance are not significant. Instead, it is probable that professional experience and professional motivation may be a driving factor in decision-making. In this case, psychiatry trainees, especially early in their training may feel more anxious in novel situations and may be less prone give a say to service users, as an attempt to avoid risks, which makes them more prone to passive SCDM [32]. However, significant differences in curricula and duration of psychiatry training across Europe [33–35] may also affect the DMS preference of trainees across European regions.

Work setting influences preference for clinical decision-making styles. Namely, working with inpatients in contrast to outpatients increases the risk of a preference for passive decision-making. In European countries where community mental health care is not yet developed, patients with most severe acute psychopathology are treated in hospitals rather than in outpatient settings. According to Hamann et al. [36], clinicians and patients in these circumstances undertake a relatively narrow spectrum of decisions—when asked about clinical decisions, inpatients with a diagnosis of schizophrenia and their psychiatrists consistently mentioned categories such as “medication,” “leave from ward/hospital,” “non‐pharmacological therapies,” and “changes in treatment setting,” in contrast to other mental health settings [36].

Finally, psychiatrists initiate involuntary hospital admission for patients with the most severe forms of acute illness (those with suicidal or aggressive behavior due to severe mental disorders) who refuse treatment. Although this in most cases is defined as a passive decision-making style, current data and European recommendations call for good clinical practice in involuntary hospital admissions as well, by raising awareness about involuntary admission procedures, patient rights, and communication about procedures [37, 38].

On the other hand, our results show that working in community mental health services is associated with the highest CDM-S score, indicating higher odds for preferring active/shared decision-making style, followed by those working in public health systems [31]. While we might assume that patients treated within community mental health teams have less severe acute psychopathology compared to inpatients, this may not be necessarily the case. This is because, in countries where community mental health teams are developed and where the number of hospital beds is very low, specialist community mental health teams may offer treatment for patients with more severe psychopathology [39–41]. In countries where community mental health teams are less developed, and care is mainly hospital-based, we may assume that hospital treatment is also offered to patients for reasons other than the severity of symptoms. Therefore, it appears that the community mental health approach may follow a different communication frame and decision-making process with patients, versus more traditional psychiatric settings. Indeed, in clinical care, SCDM is embodied within the recovery orientation in mental health care (reflected in the service users paradigm—“no decision about me, without me”) [7, 42, 43].

This is yet another argument favoring reform of mental health care services to include a community mental health care approach in European countries where these teams are still lacking.

### Limitations of the study

The study had several limitations. First, we cannot claim that the sample is representative for the relevant parameters at the country level because response rates in individual countries were low and because non-response was possibly associated with a preferred CDM style. Second, the overall number of participants is rather low, especially when it comes to the numbers in specific countries, which limits the analysis within specific countries. The sample from SEE was proportionate to its population share within Europe, but it was smaller in absolute number than the samples from the other two regions. Third, we used EuroVoc to categorize countries into regions. Such groupings are always somewhat arbitrary and may lead to grouping of countries with different characteristics. Countries within individual regions may not be homogeneous with regard to the examined outcomes. Finally, estimates of the total number of psychiatrists by country, which we used to calculate poststratification weights, were not perfect and, in addition, were not of equal quality for all countries in which we selected the sample. Given the size of the differences in the relative shares of individual countries in the sample compared to the shares in the whole population, we are reasonably certain that the impact of imperfections in our poststratification weights is markedly smaller than errors that would have followed the analysis of unweighted raw data.

### Generalisability of the results

In this study, we reported the first and most extensive set of data on decision-making style preferences of psychiatrists and psychiatry specialist trainees across Europe. Overall, we found that SDM was the preferred decision-making style across all three European regions, with on average more than 70% of decisions being rated as SDM. However, we found significant differences in the preferred non-shared decision-making styles in different European regions, mostly driven by the preference for the active style in NWE countries, and the preference for the passive style in the CEE countries. While we found differences between and within countries, as shown in Figures 1 and 2, these should be regarded only as “country snap-shots,” due to the study limitations. Overall, a passive style was specifically associated with status as a psychiatrist (versus trainee) and working with inpatients (versus outpatients), while working in a public setting, and especially in community mental health services was associated with the highest score on CDM-S, showing higher odds for preferring active/shared decision-making style across all regions.

### Implications of the findings for future practice

The study has several implications. First, this study suggests that the preference for SDM across Europe is increasing and harmonizing across Europe, which is encouraging. Second, we demonstrated differences in the non-shared decision-making styles between the three European regions, which calls for more dialogue within the European national psychiatric associations and EPA community to harmonize its practices. The EPA will use these results to promote SDM as a good clinical practice throughout Europe among mental health workers, organizations active in the field of mental health, and European policy makers.

In line with this, the EPA may promote and offer educational activities supporting the use of SDM in clinical practice at different levels: (a) continuing professional development for psychiatrists with a focus on the clinical use of PREMs and PROMs; (b) promotion of recovery-oriented practices, such as SDM and peer support in formal psychiatric training education across Europe (e.g., by incorporating this in the European Psychiatric Specialist Examination currently under development); and (c) promoting formal education on SDM at an even earlier educational stage (medical schools) [44]. Finally, the EPA should reinforce education by promoting on-site supervision, leadership, and alignment of funding resources [45].

## Data Availability

The data that support the findings of this study are available as open source.

## Supplementary material

For supplementary material accompanying this paper visit https://doi.org/10.1192/j.eurpsy.2022.2330. click here to view supplementary material
