# Supplementary material for: Clinical decision-making style preferences of European psychiatrists: Results from the Ambassadors survey in 38 countries
Source: Eur Psychiatry. 2022 Oct 21;65(1):e75. doi: 10.1192/j.eurpsy.2022.2330 (PMC9706307; doi:10.1192/j.eurpsy.2022.2330)
Supplement: Supplementary file 1 [file S0924933822023306sup001.docx]

**SUPPLEMENTARY MATERIAL**

## Calculation of poststratification weights

We determined the number of psychiatrists in each country primarily using the Eurostat 2019. Details on sources of these data are presented in Supplementary Table 1. To define countries as strata we used Stata option “poststrata” and to define poststratum population sizes we used Stata option “postweight”. We did not use a finite population correction, and we used Taylor linearized variance estimation.

**Supplementary Table 1.** Data for the calculation of poststratification weights

| Country code | Country | Region | Number of psychiatrists in country | Proportion of total European population of psychiatrists | The final sample n = 751 | Sample proportion | Poststratification weights |
| --- | --- | --- | --- | --- | --- | --- | --- |
| 1 | Armenia ^1^ | 1 | 112 | 0.000910 | 1 | 0.001332 | 0.683393 |
| 2 | Austria | 2 | 1,788 | 0.014527 | 20 | 0.026631 | 10.909880 |
| 3 | Azerbaijan ^2^ | 1 | 332 | 0.002697 | 1 | 0.001332 | 2.025772 |
| 4 | Belarus ^3^ | 1 | 1,073 | 0.008718 | 22 | 0.029294 | 6.547148 |
| 5 | Belgium | 2 | 1,960 | 0.015925 | 29 | 0.038615 | 11.959376 |
| 6 | Bosnia and Herzegovina ^4^ | 1 | 284 | 0.002307 | 14 | 0.018642 | 1.732889 |
| 7 | Bulgaria | 1 | 725 | 0.005890 | 7 | 0.009321 | 4.423749 |
| 8 | Croatia | 1 | 683 | 0.005549 | 27 | 0.035952 | 4.167476 |
| 9 | Czech Republic | 1 | 1,664 | 0.013520 | 21 | 0.027963 | 10.153266 |
| 10 | Denmark ^5^ | 2 | 1,092 | 0.008872 | 12 | 0.015979 | 6.663081 |
| 11 | Estonia | 1 | 261 | 0.002121 | 4 | 0.005326 | 1.592550 |
| 12 | Finland ^6^ | 2 | 1,293 | 0.010505 | 1 | 0.001332 | 7.889527 |
| 13 | France | 2 | 15,373 | 0.124903 | 80 | 0.106525 | 93.801779 |
| 14 | Georgia ^7^ | 1 | 265 | 0.002153 | 17 | 0.022636 | 1.616956 |
| 15 | Germany | 2 | 23,024 | 0.187065 | 29 | 0.038615 | 140.486058 |
| 16 | Greece | 3 | 2,809 | 0.022823 | 6 | 0.007989 | 17.139738 |
| 17 | Hungary | 1 | 1,487 | 0.012082 | 17 | 0.022636 | 9.073261 |
| 18 | Ireland | 2 | 944 | 0.007670 | 7 | 0.009321 | 5.760026 |
| 19 | Italy | 3 | 10,382 | 0.084352 | 26 | 0.034621 | 63.348083 |
| 20 | Latvia | 1 | 304 | 0.002470 | 22 | 0.029294 | 1.854924 |
| 21 | Lithuania | 1 | 650 | 0.005281 | 16 | 0.021305 | 3.966120 |
| 22 | Malta | 3 | 57 | 0.000463 | 2 | 0.002663 | 0.347798 |
| 23 | Moldova ^8^ | 1 | 10 | 0.000081 | 10 | 0.013316 | 0.061017 |
| 24 | Netherlands | 2 | 4,153 | 0.033742 | 3 | 0.003995 | 25.340453 |
| 25 | Norway | 2 | 1,377 | 0.011188 | 16 | 0.021305 | 8.402072 |
| 26 | Poland ^9^ | 1 | 3,504 | 0.028469 | 68 | 0.090546 | 21.380435 |
| 27 | Portugal | 3 | 1,397 | 0.011350 | 24 | 0.031957 | 8.524106 |
| 28 | Romania | 1 | 2,529 | 0.020548 | 14 | 0.018642 | 15.431256 |
| 29 | Russian Federation ^10^ | 1 | 12,202 | 0.099139 | 19 | 0.025300 | 74.453217 |
| 30 | Serbia ^5^ | 1 | 847 | 0.006882 | 17 | 0.022636 | 5.168159 |
| 31 | Slovakia ^11^ | 1 | 628 | 0.005102 | 25 | 0.033289 | 3.831882 |
| 32 | Slovenia | 2 | 323 | 0.002624 | 20 | 0.026631 | 1.970856 |
| 33 | Spain | 3 | 5,582 | 0.045353 | 57 | 0.075899 | 34.059815 |
| 34 | Sweden ^5^ | 2 | 2,383 | 0.019361 | 18 | 0.023968 | 14.540405 |
| 35 | Switzerland | 2 | 4,491 | 0.036488 | 16 | 0.021305 | 27.402836 |
| 36 | Turkey | 3 | 4,749 | 0.038585 | 41 | 0.054594 | 28.977080 |
| 37 | Ukraine ^12^ | 1 |  | 0.000000 | 0 | 0.000000 |  |
| 38 | United Kingdom | 2 | 12,343 | 0.100284 | 22 | 0.029294 | 75.313560 |
|  | TOTAL |  | 123,080 | 1.000000 | 751 | 1.000000 | 751.000000 |

Data sources:

The source if not specified otherwise: Eurostat, 2019. Available at: https://ec.europa.eu/eurostat/databrowser/view/HLTH_RS_SPEC__custom_973484/default/table?lang=en; Accessed on March 7, 2022

^1^ WHO Mental Health Atlas 2017. Available at: https://www.who.int/publications/m/item/mental-health-atlas-2017-country-profile-armenia; Accessed on March 7, 2022

^2^ WHO Mental Health Atlas 2017. Available at: https://www.who.int/mental_health/evidence/atlas/profiles-2017/AZE.pdf; Accessed on March 7, 2022

^3^ Golubeva et al. Psychiatry in the Republic of Belarus. Bulleting of the Board of International Affairs of the Royal College of Psychiatrists. 2006. 3(3):11-13

^4^ WHO Mental Health Atlas 2017. Available at: https://cdn.who.int/media/docs/default-source/mental-health/mental-health-atlas-2017-country-profiles/bih.pdf?sfvrsn=bed3d919_1&download=true; Accessed on March 7, 2022

^5^ Eurostat, 2018

^6^ WHO Mental Health Atlas 2017. Available at: https://cdn.who.int/media/docs/default-source/mental-health/mental-health-atlas-2017-country-profiles/fin.pdf?sfvrsn=24ad5cea_1&download=true; Accessed on March 7, 2022

^7^ WHO Mental Health Atlas 2017. Available at: https://cdn.who.int/media/docs/default-source/mental-health/mental-health-atlas-2017-country-profiles/geo.pdf?sfvrsn=87c9e3b8_1&download=true; Accessed on March 7, 2022

^8^ Estimated based on WHO-AIMS Report on Mental Helath System in the Republic of Moldova 2006. Available at: https://www.who.int/mental_health/republic_of_moldova_who_aims_report.pdf. Total population from WHO Mental Health Atlas 2017. Available at: https://cdn.who.int/media/docs/default-source/mental-health/mental-health-atlas-2017-country-profiles/fsm325c0ce8-b44c-4bbe-8804-43856ff0306b.pdf?sfvrsn=f11d307c_1&download=true; Accessed on March 7, 2022

^9^ Eurostat, 2017

^10^ WHO Mental Health Atlas 2017. Available at: https://cdn.who.int/media/docs/default-source/mental-health/mental-health-atlas-2017-country-profiles/rus.pdf?sfvrsn=8892c4b8_1&download=true; Accessed on March 7, 2022

^11^ Number of psychiatrists per 100,000 from WHO European Health Information Gateway. Available at: https://gateway.euro.who.int/en/indicators/hlthres_229-psychiatrists-per-100-000/. Population from wolrdometer. Available at: https://www.worldometers.info/world-population/slovakia-population/; Accessed on March 7, 2022

^12^ WHO Mental Health Atlas 2017. Available at: https://cdn.who.int/media/docs/default-source/mental-health/mental-health-atlas-2017-country-profiles/ua.pdf?sfvrsn=e834c033_3&download=true; Accessed on March 7, 2022; Ukraine was skipped from the targeted population because it was dropped off from the sample due to the missing data on all CDMS items.

**Supplementary Figure 1.** Regional distribution of number of psychiatrists in target population and the sample

**Missing data and multiple imputation**

*Methods*

We tested the hypothesis that the missing data are missing completely at random (MCAR) using Little’s test. After the strong rejection of this hypothesis, we assumed the missing data are missing at random (MAR) with the ignorable missing data mechanism, because several observed variables were relevantly associated with missingness, and because we could not think of the unobserved causes of the missing data on particular CDMS items, nor have we been able to find them in the literature. In the multiple imputation model, we included all variables that we plan to use as possible moderators of decision-making styles and all secondary outcomes.

Overall, 46/751 (6.1%) of participants had missing data on all items of the CDMS Section A but no missing data on section B, and 35/751 (4.7%) participants had missing data on all items of the CDMS Section B but complete data on Section A. The remaining missing data patterns were not monotone, but general, or arbitrary. Therefore, we conducted the multiple imputation using a sequential regression multivariate imputation or chained equations (MICE) where in the sequence of univariate imputation models all variables are used as predictors for each imputed variable. Imputations were conducted in the order from the variable with the least missing data to the one with the most of them. We included region and profession as predictors with no missing data. We used ordinal regression to impute missing data of CDMS items, frequency of appointments, access of peer support, promotion of shared decision-making in the department, and usage of PROMS/PREMS, logistic regression to impute in-or outpatients, and predictive mean matching with 5 nearest neighbours to draw from to impute duration of being qualified in years, and cost of appointment in EUR. To enable reproduction of results we set a random-number seed at 4112000.

*Results*

The finally retained participants had missing data on a maximum of 9/15 (60%) of CDMS-S items. In this final sample, there were 638/751 (85.0%) participants who responded to all 15 CDMS items. The remaining 113/751 (15.0%) participants had missing data on at least one, and not more than nine CDMS items. The proportion of missing data was almost the same in all 15 CDMS-S items, ranging from 4.9% to 6.8%. Little’s test strongly rejected the hypothesis that these missing data were missing completely at random (n = 751, Χ^2^ distance = 302.5, df = 229, p < 0.001). Therefore, we could not use only the complete cases.

**Categorization of countries into regions**

We categorized countries to three regions according to EuroVoc (Supplementary material). 1-*Central and Eastern Europe*: Albania, Armenia, Azerbaijan, Belarus, Bosnia and Herzegovina, Bulgaria, Croatia, Czech Republic, Estonia, Georgia, Hungary, Kyrgyzstan, Latvia, Lithuania, Macedonia, Moldova, Poland, Romania, Russian Federation, Serbia and Montenegro, Slovakia, Ukraine; 2-*Northern and Western Europe*: Austria, Belgium, Denmark, Finland, France, Germany, Ireland, Netherlands, Slovenia, Sweden, Switzerland, United Kingdom; 3-*Southern Europe*: Greece, Israel, Italy, Portugal, Spain, Turkey.

**Reliability and unidimensionality of CDMS-S**

*Methods*

We assessed the unidimensional reliability of CDMS-S unstandardized 15 items using McDonald’s omega (ω) reliability coefficient, and Cronbach alpha (α) coefficients of internal consistency. We tested the unidimensionality of CDMS-S using Horn’s parallel analysis and confirmatory factor analysis. As the indicators of good model fit to the observed data we used non-significant Chi-square test, comparative fit index, CFI > 0.90; root mean square error of approximation, RMSEA ≤ 0.08 with upper bound of 90% CI < 1.00, lower bound of 90% CI < 0.05, and standardized root mean square of residuals, SRMR ≤ 0.08.

*Results*

Unidimensional reliability of CDMS-S unstandardized 15 items was McDonald’s ω = 0.77 (95% CI 0.75; 0.80), and Cronbach’s α = 0.78 (95% CI 0.76; 0.80). Horn’s parallel analysis indicated existence of three relevant CDMS latent factors, and the single factor model tested by confirmatory factor analysis did not fit well the empirical data (Χ^2^ = 1274.3; df = 90; p < 0.001; CFI = 0.62; RMSEA = 0.141 (90% CI 0.134; 0.148); SRMR = 0.108). CDMS in this usage was not a unidimensional scale. First of the three latent factors extracted using minimum residual method and promax rotated was primarily measured by items B11. “If the dosage of medication should be changed” (loading 0.84), B12. “If another medication should be proscribed” (loading 0.82), B15. “How long to take the drugs for” (loading 0.74), B13 “If medication should be used at all” (loading 0.68), and B14. “In what form the drugs should be taken (e.g. depot, tablets)” (loading 0.55). This latent factor may be named “Medication”, and it explained 19.4% of manifest items variance. The second latent factor was primarily measured by items B8. “What type of occupation would be suitable (e.g. less demanding or the same as before)?” (loading 0.89), B9. “how much should the service user work (e.g. part-time or full-time)?” (loading 0.79), and B7. “If the service user can return to work” (loading 0.58). This latent factor may be named “Work”, and explained 11.0% manifest variance. The third latent factor was primarily measured by items A1. “Important treatment decisions should be made solely by the treating clinician” (loading 0.80), A3. “Decision concerning treatment in the clinic should be made solely by the hospital staff” (loading 0.74), and A2. “Service users should comply with the clinician’s advice even if they are of a different opinion” (loading 0.64). This latent factor may be named “General attitude on who should make treatment decisions” and it explained 13.2% of manifest items variance. Three latent factors in total explained 43.6% of observed items variance. The three-factors model markedly better fitted the observed data (Χ^2^ = 225.2; df = 41; p < 0.001; CFI = 0.99; RMSEA = 0.083 (90% CI 0.073; 0.094); SRMR = 0.060). The remaining four CDMS items had large uniqueness: A4. “Service users should make their own decisions concerning everyday problems related to their illness” (uniqueness 0.93), A5. “Clinicians who are treating service users should take more control when an illness gets worse” (uniqueness 0.88), A6. “The service user should decide how often they should see the clinician” (uniqueness 0.89), and B10. “If the service user should see a doctor” (uniqueness 0.83).

**STROBE Checklist for cross-sectional studies**

|  | Item No | Recommendation | Page No |
| --- | --- | --- | --- |
| **Title and abstract** | 1 | (*a*) Indicate the study’s design with a commonly used term in the title or the abstract | 1 |
|  |  | (*b*) Provide in the abstract an informative and balanced summary of what was done and what was found | 4, 5 |
| Introduction | | | |
| Background/rationale | 2 | Explain the scientific background and rationale for the investigation being reported | 6, 7 |
| Objectives | 3 | State specific objectives, including any prespecified hypotheses | 7 |
| Methods | | | |
| Study design | 4 | Present key elements of study design early in the paper | 8 |
| Setting | 5 | Describe the setting, locations, and relevant dates, including periods of recruitment, exposure, follow-up, and data collection | 8 |
| Participants | 6 | (*a*) Give the eligibility criteria, and the sources and methods of selection of participants | 8 |
| Variables | 7 | Clearly define all outcomes, exposures, predictors, potential confounders, and effect modifiers. Give diagnostic criteria, if applicable | 9, 10  S1-4 |
| Data sources/ measurement | 8* | For each variable of interest, give sources of data and details of methods of assessment (measurement). Describe comparability of assessment methods if there is more than one group | 8-10  S1, S4 |
| Bias | 9 | Describe any efforts to address potential sources of bias | 9, S3 |
| Study size | 10 | Explain how the study size was arrived at | 8 |
| Quantitative variables | 11 | Explain how quantitative variables were handled in the analyses. If applicable, describe which groupings were chosen and why | 9-10 |
| Statistical methods | 12 | (*a*) Describe all statistical methods, including those used to control for confounding | 9, 10 |
|  |  | (*b*) Describe any methods used to examine subgroups and interactions | 9, 10 |
|  |  | (*c*) Explain how missing data were addressed | 9, 10, 12  S3-4 |
|  |  | (*d*) If applicable, describe analytical methods taking account of sampling strategy | 8, S1 |
|  |  | (*e*) Describe any sensitivity analyses | x |
| Results | | | |
| Participants | 13* | (a) Report numbers of individuals at each stage of study—eg numbers potentially eligible, examined for eligibility, confirmed eligible, included in the study, completing follow-up, and analysed | 11, S1 |
|  |  | (b) Give reasons for non-participation at each stage | 11 |
|  |  | (c) Consider use of a flow diagram | x |
| Descriptive data | 14* | (a) Give characteristics of study participants (eg demographic, clinical, social) and information on exposures and potential confounders | 11, Table 1 |
|  |  | (b) Indicate number of participants with missing data for each variable of interest | Table 1 |
| Outcome data | 15* | Report numbers of outcome events or summary measures | 12, Table 2 |
| Main results | 16 | (*a*) Give unadjusted estimates and, if applicable, confounder-adjusted estimates and their precision (eg, 95% confidence interval). Make clear which confounders were adjusted for and why they were included | 12, 13, Tables 2-4 |
|  |  | (*b*) Report category boundaries when continuous variables were categorized | 9 |
|  |  | (*c*) If relevant, consider translating estimates of relative risk into absolute risk for a meaningful time period | x |
| Other analyses | 17 | Report other analyses done—eg analyses of subgroups and interactions, and sensitivity analyses | 13 |
| Discussion | | | |
| Key results | 18 | Summarise key results with reference to study objectives | 15 |
| Limitations | 19 | Discuss limitations of the study, taking into account sources of potential bias or imprecision. Discuss both direction and magnitude of any potential bias | 17-18 |
| Interpretation | 20 | Give a cautious overall interpretation of results considering objectives, limitations, multiplicity of analyses, results from similar studies, and other relevant evidence | 15-17 |
| Generalisability | 21 | Discuss the generalisability (external validity) of the study results | 19,20 |
| Other information | | | |
| Funding | 22 | Give the source of funding and the role of the funders for the present study and, if applicable, for the original study on which the present article is based | 21 |
